# To what degree are orphan drugs patient-centered? A review of the current state of clinical research in rare diseases

**DOI:** 10.1186/s13023-020-01400-0

**Published:** 2020-06-03

**Authors:** Sally Lanar, Catherine Acquadro, James Seaton, Isabelle Savre, Benoit Arnould

**Affiliations:** 1 ICON plc, Patient-Centered Outcomes, 27 rue de la Villette, 69003 Lyon, France; 2 Seaton Associates, LLC, 9039 Sligo Creek Parkway #1408, Silver Spring, MD 20901 USA; 3 Mapi Research Trust, 27 rue de la Villette, 69003 Lyon, France

**Keywords:** Patient-centricity, Patient-reported outcomes, Rare diseases, Orphan drugs, Clinical trials, Labeling claims, Patient advocacy groups, Patient engagement

## Abstract

**Background:**

Over the past 30 years, the healthcare industry has increasingly turned its attention to rare diseases. Regulators have emphasized the need for clinical research in this area to be patient-centered. However, there is a lack of evidence concerning whether this need is actually met. In this paper, we aim to address this gap.

**Methods:**

First, we describe the state of patient-centricity in clinical research in rare diseases based on a targeted literature review. Second, we discuss recommendations from scientific bodies on patient-reported outcome (PRO) measures in rare diseases. Third, we analyze data collected from EMA’s and FDA’s websites concerning rare disease labeling claims and data from Clinicaltrials.gov concerning the use of PRO measures in rare disease pivotal trials. Fourth, we perform an exhaustive literature review on the use of PRO measures in the pharmaceutical industry, including all phases of clinical research, observational/registry studies, and instrument development and validation.

**Results:**

There is limited information on rare disease patient engagement in study design, recruitment, and retention. None of the initiatives describing methods for developing PRO measures in rare diseases provide the clear guidance clinical researchers need. Only 17.4% of orphan drug labels contain a PRO measure. Less than half of pivotal trials in orphan drugs have a PRO measure as a primary or a secondary endpoint. Although the number of publications about PRO measures in rare diseases has risen in the past fifteen years, our results indicate that substantial improvements are needed to achieve patient-centricity.

**Conclusions:**

The nature and extent of patient engagement in rare disease research is under-documented. The current paradigm for developing and using PRO measures in clinical research is failing to meet the needs of rare disease patients. Not only are PROs rarely used as high-level endpoints in clinical trials or taken into account in labeling claims, they are also under-researched overall – there are too few measures for the multitude of rare diseases. We call for a clear guidance on patient engagement and suggest a realistic approach to the adaptation of PRO strategy to the specific context of clinical research in rare diseases.

## Background

### Introduction

Over the past 30 years, the healthcare industry has paid increasing attention to rare diseases and their treatments. Pharmaceutical companies, regulatory agencies, payers, and professional groups have all invested in rare diseases in different ways. There are five factors driving this trend.

The first factor is the intrinsic characteristics of rare diseases, which in and of themselves capture and hold attention. Rare disease patients often struggle for years and consult multiple specialists before receiving a correct diagnosis. Once diagnosed, very few rare diseases have effective treatments. In the absence of treatment, many rare diseases which are progressive and/or life-threatening lead not only to a loss of physical and cognitive capabilities but also to premature death [1].

The contrast between the large number of rare diseases and the small number of rare disease patients is the second factor driving the healthcare industry’s investment in this area. Due to new descriptions of rare subtypes of existing diseases and improvements in diagnostics and understanding of disease pathology, the already substantial number of rare diseases is likely to increase: each year, 250 to 280 new rare diseases are identified, adding to the known 6000 to 8000 rare diseases [1]. The quantity of rare diseases needs to be considered in relation to the small number of rare disease patients: at the worldwide level, the estimated population prevalence of rare diseases is 3.5–5.9%, or 263–446 million persons affected at any point in time [2].

The third factor is the adoption by key institutions of policies providing incentives and support to researchers and industry in the field of rare diseases. Legal and financial policies instituted since the 1980s, such as the United States Orphan Drug Act in 1983 and the European Union Regulation on Orphan Medicines in 2000, laid the groundwork for value-based healthcare opportunities that reward innovation when unmet patient needs are high, as is the case with rare diseases. Indeed, rare disease treatments are receiving increasing numbers of orphan drug designations and market authorizations, so much so that they are predicted to account for one-fifth of all prescription drug sales by 2024 [3].

The fourth factor is the information-sharing and information-seeking behaviors of rare disease patients via online communities, forums, and search engines [4]. In recent years, these communities have begun to take on a major role in the pharmaceutical industry space, leveraging their connections with patients and physicians as a way to demand treatments and measurement strategies that reflect their needs and goals [5].

The fifth and final factor is the strength of rare disease patient advocacy organizations (PAOs). PAOs are active throughout the medical drug life cycle, from early stage research to clinical trials to commercialization. One recent example of the strength of PAOs is the Parent Project for Muscular Dystrophy’s collaboration with patients, disease experts, pharmaceutical companies, and external consultants to develop a draft FDA guidance for the healthcare industry [6]. Another example is the work of the Cystic Fibrosis Foundation to identify the shared priorities of researchers, patients, and families [7].

These five factors lay the groundwork for a healthcare industry that, in theory, would be developing and marketing medications that address some of the needs that are most important to rare disease patients and their families. The goal of the present paper is to examine whether this situation is currently the case and, if it is not, to propose possible explanations and solutions.

To this end, we will use a number of terms that need to be defined because they may be ambiguous or have multiple meanings [8–11]. In this paper:

- “Patient”: is used as a shorthand for rare disease patients themselves, their families and caregivers, and PAOs
- “Patient-centricity”: is used to describe actions “putting the patient first in an open and sustained engagement of the patient to respectfully and compassionately achieve the best experience and outcome for that person and their family” [12]
- “Patient engagement”: is used to describe actions taken by patients alone or in coordination with researchers which aim to influence the research agenda, the design of studies, and the decisions of stakeholders (regulators and payers)
- “Patient-reported outcome” (PRO): is used to describe a “report that comes directly from the patient [ …] about the status of the patient’s health condition without amendment or interpretation of the patient’s response by a clinician or anyone else” [13]. The term patient-reported outcome measures (“PROM”) relates to the instrument used to capture a PRO

In our paper, we assume that all forms of patient engagement and all PROMs are patient-centric. We recognize that this is not always the case. If not well developed, validated, or selected, PROMs may not be relevant to patients who complete them. Similarly, patient engagement can be tokenistic when it is done only at a superficial level. However, for practical purposes, we have made this assumption because it allows us to examine as a cohesive whole three levels at which the patients’ voice may impact clinical research. The first level is the strategic level: when patients contribute to study design or interact with regulatory bodies. The second level is the endpoints level: when patients complete a PROM in a study or participate in the development and validation of a PROM. The third level is the participatory level: when patients work with researchers to improve recruitment and retention.

The first section of this paper will focus on what the available literature can tell us about the impacts patient engagement can have on the strategic and participatory levels of clinical research in rare diseases. The second section will focus on patient-centricity at the endpoints level; in other words, it will cover the development, validation, and use of PROMs in rare disease clinical research. The role of PROMs will be analyzed in the context of (1) labeling claims, (2) outcome measures of pivotal trials, and (3) the larger pharmaceutical industry space, including all phases of clinical research, observational/registry studies, and instrument development and validation. In conclusion, we will discuss possible causes driving our findings, gaps remaining in achieving patient-centered orphan drug development, and solutions for closing these gaps.

## Main text

### Patient engagement in rare disease clinical research

#### Limitations in the published literature on this topic

We found it difficult to ascertain the breadth and depth of rare disease patient engagement in clinical trials because at the moment research on this topic is limited in three areas.

First, there are no best practices, guidelines, or metrics for stakeholders to use to select their activities and to assess the success or failure of these activities. Indeed, even basic vocabulary in the field is ambiguous. Words like “patient engagement,” “empowerment,” and “involvement” are often used without being fully defined [8–11]. None of the efforts made to define and differentiate terminology [8, 10] have gained widespread acceptance.

Second, published articles describing patient engagement are unlikely to distinguish among the contexts in which that engagement takes place, e.g., study type, study objectives, and disease areas [11, 14]. Therefore, reliable comparisons cannot be made between types of patient engagement. For example, a comparison of patient engagement involving recruitment and retention vs. patient engagement involving study design cannot be performed with the available data.

Third, articles on patient engagement are limited in terms of their source data or have no source data at all. Some articles only review studies that received a certain source of funding [14–17] or only use data available to a particular organization [18, 19]. Many articles not based on original research nonetheless include broad recommendations. The generality of these recommendations makes them difficult to implement in the case of rare diseases that require tailor-made solutions by disease area and by study objective [8, 10, 20, 21]. To the best of our knowledge, there is only one systematic literature review, by Forsythe et al.*,* published in 2014, on the topic of patient engagement in clinical research in rare diseases [9].

#### Patient engagement in rare disease study recruitment and retention

Despite the limitations noted above, the published literature does allow us to have some perspective on how patient engagement influences the participatory aspect of clinical research in rare diseases.

First, it is clear from the literature that rare disease PAOs are crucial for connecting drug researchers with patients who are potential participants in clinical trials. Published studies “frequently acknowledge the role of rare disease PAOs in providing access to or recruiting patients both for engagement as study subjects and in enhancing communication about research participation opportunities and findings” [9]. In Europe, a EURORDIS survey of member organizations’ research support initiatives, conducted from October to November 2009, found that of the 309 patient organizations that responded, more than half (57%) identified patients to participate in clinical trials and slightly less than half (49%) provided information and counselling to trial participants [18]. In the United States, PAOs play a similarly active role in trial recruitment and retention. A 2008 survey of 124 PAOs that were members of the Genetic Alliance at that time found that 91% of organizations had helped to recruit research volunteers [19]. Another survey conducted in 2016 [15] by the Rare Disease Clinical Research Network (RDCRN) found that PAOs contributed more than 40% of the total number of patients enrolled in trials run by consortia members. The importance of PAOs was highlighted both by Principal Investigators (PIs) running the trials and PAOs alike, with 16 of 17 consortium PIs surveyed reporting this type of interactions and 24 of 28 PAO representatives reporting it. PAOs involved in the RDCRN increased awareness about trial participation via their websites and supported group meetings where they shared information on trials and answered questions. They were also the main source of patient referrals to the Patient Contact Registry, a database of patients who are interested in participating in research that the RDCRN manages. In 2019, the U.S. National Organization for Rare Disorders (NORD) and the Critical Path (C-Path) institute launched a joint program to collect, aggregate, analyze, and store data from patients and PAOs. This program aims to enable faster understanding of disease progression and development of appropriate clinical trial designs [22, 23].

While working with PAOs to recruit patients does not, per se*,* make a study patient-centric, when sponsors *do* work through PAOs, patients are more likely to trust that their choices concerning enrollment will not influence the care they receive from their clinician. When clinicians invite them to participate in a trial, some patients may fear that declining will disappoint their clinician and reduce their clinician’s investment in their care. By contrast, patients are less likely to feel that declining an invitation from a PAO carries a comparable *personal* risk of reduced attention from their clinician. Furthermore, when several trials compete for patients, clinicians acting as trial investigators may not be in the best position to provide patients with neutral information. PAOs, however, can take an impartial stance and help patients understand the advantages and disadvantages of each trial.

Second, the literature describes efforts designed to make study procedures easier for patients, which in turn may improve recruitment and retention. To this end, Mullins [24] and Potter [20] recommend considering pragmatic, Bayesian, and adaptive trial designs. These types of trials recruit from a larger variety of locations, have less stringent inclusion criteria, and are likely to include patient-centered outcomes. As a result, they better represent both real-world conditions and the concerns of patients. Bayesian statistical analyses allow for continual modifications of trial design as more information becomes available, such as changing sample sizes, adding/removing treatment arms, and ending the trial altogether if results are unpromising. These efforts respond in part to the logistic and emotional concerns that patients may have about clinical trial procedures. Such concerns may deter them from participating in clinical trials and/or lead them to drop out along the way.

Even patients who are motivated to help find a cure for their disease (especially minor children or adults with impaired mobility) may choose not to participate because the logistics of participation — e.g., travel to a far-away clinic, frequent medical tests, incompatibility with job requirements or with other medications, etc. — make it too burdensome. Patients may also be concerned about how participation could influence their lives in the future. For instance, patients may worry about the long-term consequences of receiving the drug being studied, or, if they are eager to try the drug, may worry that they will receive the placebo instead; they may also fear potential financial burdens or negative health impacts if the trial produces unintended harm. The choice to not only enroll but to *stay* enrolled can be an even more complex and emotionally-charged decision when patients do not trust sponsors to act in their best interest. For example, patients who doubt that sponsors will fully and accurately inform them of potential adverse events may be less likely to enroll and more likely to drop out.

The following are some recommendations that may, at least partially, address patients’ logistical and emotional concerns:

- Design studies to reduce or eliminate burdensome study requirements
- Take proactive steps to acknowledge patients’ feelings about study participation
- Keep study participants fully and honestly informed about the study’s progress and its impact on patients
- Emphasize sponsors’ interest in obtaining the best possible outcome for each patient
- Build trusting relationships with PAOs who already have patients’ confidence

Although recruiting and retaining patients for clinical trials is a challenge regardless of the disease area, the small number of rare disease patients creates a context of competitive recruitment that raises the stakes for both sponsors and patients. Whereas in more common diseases, one patient declining to enter a trial or withdrawing mid-way through may have little impact on the overall study outcome, in rare diseases this patient may be one of only of a handful of patients worldwide with the diagnosis being studied. Likewise, although many trials or treatment options may be available for a common disease, only one trial may be enrolling at any given time for patients with a rare disease. In such cases, when the consequences of each individual patient’s decision to enter and/or remain in a clinical trial may be key to both the trial’s success and the patient’s health, it may be useful for both sponsors and patients alike to work with PAOs and to follow some of the recommendations given above.

#### Patient engagement in rare disease study design

In addition to adopting targeted recruitment and retention strategies, some sponsors have actively engaged rare disease patients in study designs. Generally, sponsors have sought advice from PAOs acting on patients’ behalf to enlist the help of patients in this area. The EURORDIS survey of member PAOs [18] as well as the Genetic Alliance survey of member PAOs [19] both found that, of the PAOs surveyed, more than half had advised researchers on study design. Even higher levels of engagement were found in the RDCRN study [15]: 82% (14 out of 17) of PIs reported that patients reviewed protocols and were “providing substantive input on study design” and 68% (21 out of 28) of PAOs reported this activity.

The systematic literature review published in 2014 by Forsythe et al. on patient engagement in rare diseases describes in more detail how patients and their representatives (PAOs and caregivers) contribute to study designs [9]. The authors reviewed 35 articles about patient engagement. Six of 35 articles mentioned patients contributing to the identification of relevant outcomes and/or the development of new measures. Five articles reported patients helping to make study designs more patient-centric. Eleven articles reported patients contributing via workshops, focus groups, or Delphi methods. The authors also described patients as sitting on governing bodies or advisory committees (five out of 35 articles), developing study documents (four out of 35), and collecting data by interviewing (also four out of 35). Despite the responsibilities of these engagements, only four studies trained patients for this role and no studies trained sponsors for their interaction with patients.

This absence of training was one of the challenges confronting patient engagement that Forsythe et al.’s 2014’s systematic literature review identified. Other challenges included insufficient time and resources (both financial and logistical) devoted to patient engagement and to helping patients overcome health hurdles that reduce their availability to contribute to study designs. The authors also raised the concern that patients who are involved in study designs may have needs and/or assets that are not representative of a given rare disease’s patient population. For their part, PAOs acting on behalf of patients may have conflicts of interest with industry when their organization receives funding directly from the study sponsor [25].

A mixed-methods study of rare disease patient organizations in Australia [26] describes other types challenges PAOs face when working on study design. PAO leaders said that their teams often did not have means and the skills needed to participate actively in research decisions. Some leaders considered that this lack of expertise made researchers unwilling to consider patients as equal partners and to give their knowledge the same credence as that of medical professionals. Other PAO leaders took a more balanced viewpoint, stating that it was sometimes difficult to find an appropriate place for the patient viewpoint in the current industry climate. It should be noted that at the time of the article’s publication Australia did not have a nation-wide policy framework for rare diseases like the US or the European Union. Therefore, some of the struggles of Australian PAOs could be due to the absence of such a framework. That said, other studies on non-rare disease patient engagement in the United States echo the opinions that Australian PAO leaders expressed [9, 11].

None of the studies included in Forsythe et al.’s 2014 systematic review on the subject explained how patient-sponsor relationships were formed or evaluated the extent of patient engagement in study design. Indeed, of the 35 studies, 28 were classified as “minimally descriptive” and only seven as “sufficiently descriptive for others to replicate” [9]. Given this absence of detail in the literature, sponsors have limited resources to consult on how to build a successful partnership with patients. While patients and sponsors could look towards common conditions for examples of patient engagement, it is not always possible to draw parallels between common conditions and rare diseases. Granted, some aspects of patient engagement are similar in both areas, such as a lack of bi-directional communication and insufficient documentation of the impact patient engagement has on study results. That said, rare disease patients, unlike their counterparts in more common diseases, are often neither trained in engagement techniques nor have knowledge about how to correct for their own potential biases [9].

Our results demonstrate that the available literature does not sufficiently explain how to best work with rare disease patients on study design, recruitment, and retention. That said, recent developments suggest that this situation is starting to change [27]. PAOs, like the International Fibrodysplasia Ossificans Progressiva Association, and international rare disease organizations, like EURORDIS, are releasing guidances on this topic. Coalitions of sponsors, such as the European Federation of Pharmaceutical Industries and Organizations and the Pharmaceutical Manufacturers of America, are following suit. However, an industry-wide guidance drafted by a regulatory agency such as the FDA or the United Kindgom’s National Institute for Health Care Excellence (NICE) is missing from the current landscape. Such an industry-wide guidance would help guarantee the consistent use of best practices and encourage sponsors and PAOs alike to adhere to its contents.

### PROMs in rare disease clinical research

#### Adapting PROMs for use in rare diseases

The growing interest in rare diseases from a regulatory and industry perspective accentuates the need for creating and/or selecting appropriate PROMs for this population. Various bodies in the healthcare industry have addressed this issue. The FDA draft guidance on rare diseases [28] gives recommendations on PROMs that are similar to those for common diseases [13]. These include considering the choice of PROMs early in the research process and setting aside time and resources, if needed, for the modification of existing PROMs and/or the development of new ones. The FDA recommends that PROMs should be evaluated in terms of their validity, reliability, feasibility, and ability to detect change. The FDA notes that given the heterogeneity of rare diseases, sponsors should consider the fact that the characteristics of PROMs may differ depending on the patient sub-population.

The International Rare Disease Research Consortium (IRDiRC) published its own report and recommendations on this subject in 2016 [5, 29]. The first part of the report summarizes the history and regulations around patient-centered outcomes (PCOs) in general, not just PROMs. The second part discusses PCOs in the specific context of rare diseases, noting that members of the rare disease community, of which small biotechnology companies and PAOs are part, often do not have the funds or the awareness needed to develop new outcome measures when they are required. The third part details the recommendations of the draft ISPOR Task Force on PROMs in rare diseases [30] (which we review in full below) and provides a list of resources focused on outcome measures for rare diseases. The final section of the IRDiRC report contains recommendations, with an emphasis on qualitative research. In order to reduce time and costs, the report advises using qualitative research to select an outcome measure with strong content validity or to adapt an existing outcome measure. If no appropriate outcome measures exist, the report’s authors argue that researchers should perform exploratory interviews with patients and other stakeholders to produce a conceptual model that will serve as the basis for the development of a new fit-for-purpose outcome measure.

Although the FDA guidance and the IRDiRC report are useful, at the moment the most in-depth source of best practices available for PROMs in rare disease clinical trials is the ISPOR Task Force report on this subject [30]. Using the FDA roadmap for outcome measures [31] as a framework, the report addresses the three steps of PROM development (understanding the disease/condition; conceptualizing treatment benefit; selecting/developing the outcome measure) through the lens of rare diseases. For each step, the authors identify challenges and suggest solutions. The challenges described originate from (1) the small numbers of patients with rare diseases, (2) the heterogeneity of clinical manifestations and of disease progression, and (3) the incapacity of certain patients to complete questionnaires without assistance.

To account for the small numbers of patients with rare diseases, the Task Force suggests working with PAOs to support recruitment and preparation of study materials. Concept elicitation interviews – the first step in PROM development – could be done with individuals other than the patients themselves, such as members of PAOs, caregivers, family members, friends, and school teachers. The same individuals could participate in concept elicitation interviews and cognitive interviews, thereby reducing the total number of people needed. Alternative types of statistical analyses are also suggested for dealing with underpowered measures.

To guarantee that a PROM covers the heterogeneity of a given rare disease, the Task Force emphasizes the need to use diverse data sources and not to exclusively rely on patient interviews. Potential data sources include: interviews with medical experts and key opinion leaders, case studies, natural history studies, medical record data, policy papers, and online blogs and forums. When analyzing data, the Task Force recommends focusing on changes over time in disease presentation, because disease severity is likely to vary over the course of patients’ lives. Geographical and cultural differences in disease experience should also be considered to ensure the international content validity of tools used. Finally, analyses should aim to extract the most over-arching symptoms and impacts so as to develop comprehensive measures.

Young children, patients with limited cognitive capacity, and patients who are very ill may be incapable of completing PROMs alone. In this situation, the Task Force recommends using observer-reported outcomes and clinician-reported outcomes. Sponsors should keep in mind however that observer-reported and clinician-reported outcomes are measurements that originate from two different populations (observers and clinicians) and therefore cannot be combined to form a single endpoint.

Although the Task Force report is a starting-point for discussing a challenging problem in rare disease clinical research, it has several limits [32]. First, given the international context of medical products and the diverse requirements of national health technology assessment bodies, the FDA roadmap can be considered a somewhat narrow lens for examining the issue. Furthermore, while the FDA roadmap as a general framework makes sense in orphan-drug development, its strict application may restrict the ability of researchers to invent the creative solutions needed to address measurement challenges in the context of rare diseases. Last, exploring alternative statistical approaches for psychometric validation and strategies that take into account payer needs would also broaden the applications of the report.

#### PROMs in the labeling claims for rare diseases

##### Search strategy

The role of PROMs in the FDA and the EMA evaluations of orphan drugs provides insight into the importance regulatory agencies give to these measures. To this end, we conducted searches of the FDA and the EMA websites on July 24, 2017 to retrieve all products approved with an orphan drug designation from January 1, 2002 to June 30, 2017, inclusive. We chose the year 2002 as a starting point as it coincided with the date of the EMA’s first approval of an orphan medicine.

We explored the FDA Orphan Drug Product designation database using the above dates as search criteria and limited the results to approved products. We reviewed the Human Medicine section of the EMA website according to type of approval and selected orphan medicines among the six possible choices (i.e.*,* additional monitoring, generics, biosimilars, conditional approvals, exceptional circumstances, and orphan medicines). We downloaded an Excel file from both website databases with all search results.

We analyzed the FDA label and the EMA summary of product characteristics of all products to find any indications of PROMs sponsors used that the EMA and the FDA considered relevant. If needed, we reviewed the corresponding FDA medical reviews or EMA assessment reports to clarify endpoint positioning and the name of the PROM used.

##### Results

For the FDA, the search retrieved 410 orphan drug product designations, representing 298 different products (i.e.*,* distinct new drug applications and biologics license applications). For the EMA, the search retrieved 101 products for a total of 119 different indications and designations (some products corresponded to more than one indication/designation). The review of both datasets showed that the EMA had 42 designations not included in the FDA dataset. This led to a combined data set of 452 distinct designations. After the 194 products with an indication for oncology were excluded, a total of 258 designations were left to review. We did not include rare cancers in our scope because technically their definition is based on incidence and not on prevalence [2]. Furthermore, their diagnosis, impact, course, and treatment are in general closer to common cancers than to non-cancer rare diseases.

The review of these 258 designations showed that only 45 designations (17.4%) included PROMs in their labeling claim. These 45 designations represented 42 different products of which 10 were common to both the FDA and the EMA. The PROMs described in the label primarily focused on symptoms (e.g., dyspnea, fatigue, pain) and rarely examined functioning or health-related quality of life (HRQoL). The majority of the labeling claims contained study-specific symptom scales (*n* = 26) or legacy, generic measures (*n* = 10). Only nine labeling claims were for rare disease-specific PROMs. In these nine labeling claims, six rare disease-specific PROMs were found (some rare disease-specific PROMs were found in more than one labeling claim) [33].

#### PROMs used in pivotal clinical trials for rare diseases

##### Search strategy

To assess how frequently PROMs were used in pivotal clinical trials in rare diseases, we searched the Clinicaltrials.gov database on July 1, 2017 with the key words (“rare” AND “disease”) OR “orphan” and selected “Interventional Studies” and “Phase III.” We did not apply any restrictions on study status. The time frame searched was from January 1, 2002 (the year of the first approval of an orphan drug by the EMA) to July 1, 2017. The search retrieved 238 results that we reviewed in order to determine if they met the criteria of being related to a rare disease and/or an orphan product. Of these 238 results, we excluded *n* = 123, including:

- Studies which were not about a rare disease and/or orphan product but had nonetheless been found by the search because they contained terms similar to the keywords (*n* = 86)
- Studies with a status of withdrawn or an unknown status (*n* = 17)
- Studies which were for products for malignant neoplasms (*n* = 20)

The final number of studies which were included and evaluated in detail was *n* = 115.

##### Results

**Fig. 1:**
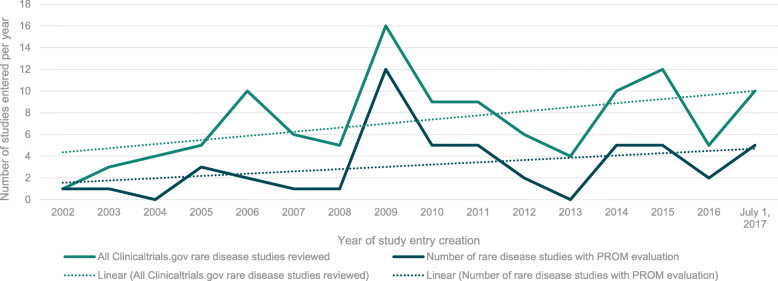
Clinicaltrials.gov entries from January 1, 2002 to July 1, 2017: Rare diseases overall vs. rare diseases with a PROM evaluation

First, we examined the trend in change over time from 2002 to 2017 in the use of PROMs in rare diseases studies. To do so, we classified the 115 studies into two groups: (1) all studies vs. (2) studies with a PROM evaluating any concept. We then sorted studies annually from 2002 to 2017 according to the date of creation of the Clinicaltrials.gov study entry. The results showed that the number of studies increased overall in both groups during this time period (Fig. 1). Of the 115 studies, less than half (*n* = 50, 43.5%) had a PROM in their study design.

The results of this analysis also suggest a possible impact of new regulatory guidance on the use of PROMs in rare disease clinical trials. The peak of 12 rare disease studies with a PROM in their study design occurs in 2009, the same year as the release of the FDA guidance on the use of PROMs in clinical trials [13]. After 2009, the number of PROMs in rare disease study designs decreased until 2014. In 2014, the number increased to five studies and in the next three-and-a-half years remained at levels higher than those before 2009. The cause of the decline from 2009 to 2013 is uncertain. While it could partially be attributed to the corresponding decline in the total number of pivotal rare disease trials, this amount does not fall below four per year in 2013. Further research is needed to elucidate the reasons behind this decline.

Second, we looked at whether a pivotal trial had a PROM in its study design and if so, what concepts that PROM was meant to assess (see Table 1). We extracted this information from the pivotal trial’s Clinicaltrials.gov entry. Of the 50 studies with PROMs, 29.6% (*n* = 34) had PROMs which measured the concept “Symptoms” and 19.1% (*n* = 22) had PROMs which measured the concept “HRQoL.” Other concepts assessed by PROMs included “Health status/perceived general health,” “Functioning,” “Impact,” “Rescue medication,” “Global impression of change,” “Ease of use,” “Compliance,” and “Disease activity.”

**Table 1:** Concepts measured by PROMs included in rare disease studies

Concept assessed by PROMs	Number of studies with a PROM measuring this concept	% of total (out of 115 studies)
Symptoms	34	29.6
Health-related quality of life	22	19.1
Rescue medication	10	8.7
Functioning (activities of daily living, disabilities)	5	4.3
Health status, perceived general health	4	3.5
Impact (e.g., of fatigue, disruptions at work/school)	3	2.6
Global impression of change	3	2.6
Compliance	2	1.7
Measure of ease of administration of a device	1	0.9
Disease activity	1	0.9

Source: Clinicaltrials.gov database search on July 1, 2017

Third, we looked at the outcome rankings in the study designs of the 22 rare disease studies that included PROMs assessing the concept “HRQoL.” We chose to focus exclusively on studies with PROMs assessing the concept of “HRQoL” because we wanted to examine how often sponsors chose to measure the impact of a symptom instead of the presence, severity, or frequency of a symptom. Of the 18 PROMs included in these 22 studies, only one, the *Acromegaly Quality of Life Questionnaire*, was listed on Clinicaltrials.gov as a primary outcome. All other PROMs were listed as secondary outcomes (see Table 2).

Fourth, in order to determine the extent to which the 22 studies using PROMs measuring “HRQoL” employed questionnaires that were fit-for-purpose, we analyzed PROMs depending on whether they were rare-disease specific, non-rare disease specific, generic, or study-specific (see Table 3 for category definitions and examples of these four categories). We then sorted the 22 studies according to the category that best fit the PROM included in the study design (see Table 2).

**Table 2:** PROMs measuring the concept of “HRQoL” in rare diseases: outcome rankings in study designs and disease specificity

Name of PRO questionnaire measuring concept of “HRQoL”	# of studies including this PROM	Study code in Clinicaltrials.gov	Study start date (YYYY/MM)	Therapeutic Indication	Primary outcome	Second-ary outcome	Rare disease specific PROM	Non-rare disease specific PROM	Generic PROM	Study-specific PROM
Acromegaly Quality of Life Questionnaire (AcroQoL)	1	NCT02952885	2017/01	Acromegaly	1		1			
AddiQOL	1	NCT01063569	2010/02	Addison’s Disease		1	1			
Children’s Dermatology Life Quality Index (CDLQI)	1	NCT02860494	2017/01	Facial Angiofibromas		1		1		
Dermatology Life Quality Index (DLQI) adults	2	NCT02383589	2015/05	Pemphigus Vulgaris		1		1		
		NCT02860494	2017/01	Facial Angiofibromas		1		1		
Euroqol EQ-5D (EQ-5D)	2	NCT02839642	2016/05	Progressive Supranuclear Palsy		1			1	
		NCT03023540	2017/03	Charcot-Marie-Tooth Disease, Type IA		1			1	
Hemophilia-Specific Quality of Life Index for Adults (Haem-A-QoL) Questionnaire	2	NCT01027364	2009/12	Severe Hemophilia B		1	1			
		NCT01181128	2010/11	Severe Hemophilia A		1	1			
Hemophilia-Specific Quality of Life Index for Children (Haemo-QoL) Questionnaire	1	NCT01027364	2009/12	Severe Hemophilia B		1	1			
Kansas City Cardiomyopathy questionnaire (KCCQ)	1	NCT02590601	2016/01	Peripartum Cardiomyopathy		1		1		
Minnesota Living with Heart Failure Scale	1	NCT01416636	2009/03	Non-operable Chronic Thromboembolic Pulmonary Hypertension		1		1		
Not specified	3	NCT00376077	2005/08	Immune Thrombocytopenic Purpura		1				1
		NCT00811681	2008/12	Friedreich’s Ataxia		1				1
		NCT01327274	2011/12	Pectus Excavatum		1				1
Patient Global Assessment of HRQL	1	NCT01940094	2014/02	Granulomatosis With Polyangiitis		1			1	
PedsQOL	3	NCT00432744	2007/01	Mitochondrial Diseases		1			1	
		NCT01197378	2010/08	Cystinosis		1			1	
		NCT01268033	2010/12	Childhood Idiopathic Nephrotic Syndrome		1			1	
PROMIS	1	NCT01940094	2014/02	Granulomatosis With Polyangiitis		1			1	
PSP-Quality of Life Scale	1	NCT02839642	2016/05	Progressive Supranuclear Palsy		1	1			
SF-36	7	NCT00294671	2006/02	Familial Amyloid Polyneuropathy; Familial Amyloidosis		1			1	
		NCT00879229	2009/07	Idiopathic Pulmonary Fibrosis; Pulmonary Hypertension		1			1	
		NCT01063569	2010/02	Addison’s Disease		1			1	
		NCT01197378	2010/08	Cystinosis		1			1	
		NCT01291433	2011/03	Radiation Induced Brachial Plexopathy		1			1	
		NCT01940094	2014/02	Granulomatosis With Polyangiitis		1			1	
		NCT02106520	2014/04	Hereditary Hemorrhagic Telangiectasia; Epistaxis		1			1	
St. George’s Respiratory Questionnaire (SRGQ)	1	NCT00879229	2009/07	Idiopathic Pulmonary Fibrosis; Pulmonary Hypertension		1		1		
The Behçet’s Disease Quality of Life (BD-QoL)	1	NCT02307513	2014/12	Behçet Syndrome		1	1			
World Health Organization (WHO) quality of life questionnaire (WHOQOL-BREF)	1	NCT02590601	2016/01	Peripartum Cardiomyopathy		1			1	
**Total**^**a**^	**31**	**–**	**–**	**–**	**1**	**30**	**7**	**6**	**15**	**3**

^a^ The total number of studies including PROMs is 31 and not 22 because some studies included more than one PRO in their study design

Source: Clinicaltrials.gov database search

**Table 3:** PROM categories, definitions, and examples

PROM category name	Definition	Example(s)
Rare disease-specific	Developed for a particular rare disease and used in a trial for that disease	*Hemophilia-Specific Quality of Life Index* for Children Questionnaire used in a trial for children with hemophilia
Non-rare disease- specific	Developed for a disease which is not a rare disease, but used in a rare disease trial	*Dermatology Life Quality Index* used in trials for adult patients with pemphigus vulgaris or facial angiofibroma
Generic	Developed for generic use and used in a rare disease trial	*SF-36* used in trials for adults with Addison’s disease or *EQ-5D* used in trials for adults with progressive supra-nuclear palsy
	Includes generic measures used in populations of children and the elderly	
		*PedsQL* used in trials for children with mitochondrial diseases, cystinosis, or childhood idiopathic nephrotic syndrome
Study-specific	A measure whose name is not provided or is defined as study-specific	Quality of life changes over time and between the treatment groups used in trials with patients with immune thrombocytopenic purpura

The results presented in Table 2 demonstrate that in terms of study design, generic questionnaires are most often used to measure “HRQoL” in rare disease clinical trials (*n* = 15 studies out of *n* = 22 total). The number of generic questionnaires is more than the twice that of rare disease-specific questionnaires (*n* = 7 studies). That said, fit-for-purpose rare disease-specific questionnaires are used more often to measure “HRQoL” than non-rare disease-specific (*n* = 6) or study-specific questionnaires (*n* = 3). Furthermore, in half of the studies with more than one PROM assessing “HRQoL,” there is a pairing of a generic PROM with a rare-disease-specific PROM or a disease-specific PROM (*n* = 4 of the 8 studies with more than one PROM). Of the remaining four studies with two PROMs included in the study design, three have PROMs measuring “HRQoL” for both adults and children (studies in Facial Angiofibromas, Severe Hemophilia B, and Cystinosis). Only one study with two PROMs included in the study design (in Granulomatosis with Polyangiitis) has multiple generic PROMs for adults measuring “HRQoL.”

#### PROMs for rare diseases which exist or are in development

##### Search strategy

In this section, we are interested in:

- PROMs used in clinical trials of all phases of drug development (whether randomized or not)
- PROMs used in observational studies or registries
- PROMs that are in the process of being developed and/or validated or have already been developed and/or validated but have not yet been used in a clinical trial, observational study, or registry.

To identify all relevant PROMs that fit this description, we performed a literature review using the OvidSP interface of the bibliographic databases Medline, Embase, and The Cochrane Central Register of Controlled Trials. We designed the search strategies for Medline and modified them for Embase and The Cochrane Library. We included free text terms as well as controlled terms from Mesh in Medline and Cochrane and Emtree in Embase. We limited searches to results in the English language with an abstract published in the last 15 years.

The literature searches we performed on June 21, 2017 in Medline, Embase, and Cochrane retrieved 1023, 1319, and 92 references, respectively. We exported the references from all three searches to a reference manager software (EndNote X7). After we removed the duplicates, 1884 articles remained. We screened these 1884 articles based on titles and abstracts. We considered articles about observational/registry studies, clinical trials of all phases (both randomized or not), and questionnaire development and/or validation. We retained the article if it (1) focused on patients with rare diseases and (2) its abstract and/or title had the name(s) of one or several PROMs. We excluded articles about case reports, chart reviews, diagnoses, qualitative research, in-vitro studies, and genetic studies.

As the terms “rare disease” and “PROM” have a variety of uses, we defined how these terms would be understood in our screening process. For rare diseases, when a rare disease was identified as such in the abstract/title, we consulted Orphanet’s website, the portal for rare diseases and orphan drugs, to confirm that the rare disease in question was indeed listed in Orphanet’s database. If a disease was identified as a rare disease in the abstract/title, but was not listed in Oprhanet’s database, Orphanet was contacted to ask for their input. We excluded articles about diseases that Orphanet said were not rare diseases according to their categorization. We included articles about diseases not yet in Orphanet’s database but for which the organization said it had future plans to evaluate them.

For PROMs, when a measure was described in the abstract/title, we assessed whether it was a PROM in one of two ways. Either (1) it was clearly specified in title/abstract (by the use of terms such as “self-administered,” “self-reported,” or “patient-reported”) that the measure was a PROM or (2) we checked in the PROQOLID™ database (through the ePROVIDE platform) for information about the development and/or validation of the measure or for a review copy of the measure. We included self-administered study-specific questionnaires and composite measures including a PROM evaluation. We excluded PROMs completed by caregivers/parents.

**Fig. 2:**
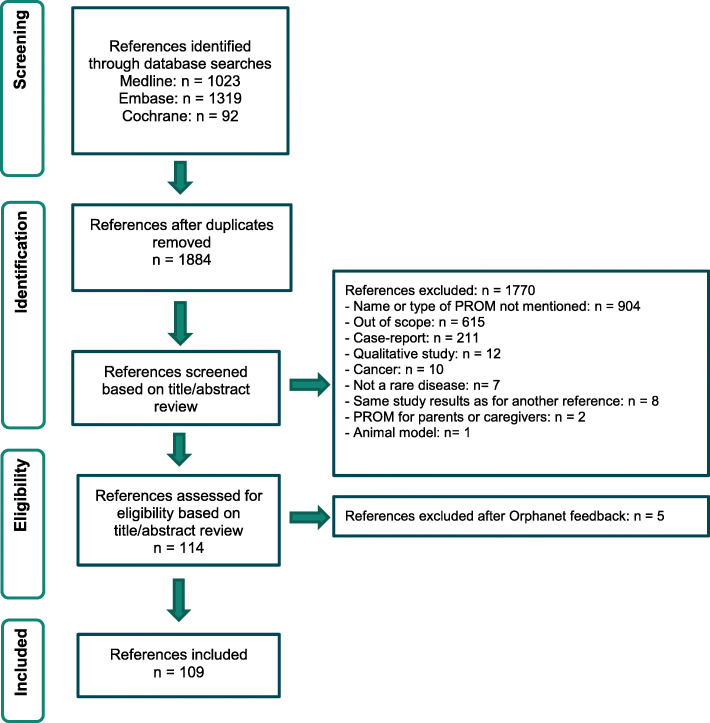
PRISMA diagram for literature review search on rare disease PROMs which exist or are in development

Overall, from the 1884 references identified in the databases searches, we extracted 109 articles about a PROM used in rare diseases. Figure 2 illustrates the PRISMA diagram of our search. We extracted names and acronyms of all identified PROMs along with data for the following variables:

- The therapeutic indication in which the measure was used
- The concept the PROM measured
- The category of the PROM: rare disease specific, non-rare disease specific, generic, or study-specific (see Table 3 for definitions)
- The context of use of the PROM: (1) used in clinical trials of all phases (whether randomized or not), (2) used in observational studies or registries, and (3) in the process of being developed or validated or have already been developed or validated but have not yet been used in a clinical trial, observational study, or registry

We identified the concepts each PROM measured according to the following process:

- If the concept(s) was specified in the abstract/title, we used this information
- If the concept(s) was not specified in the abstract/title, we searched for the PROM in the PROQOLID™ database and extracted the concept(s) listed in the database
- If the PROM concept(s) was not reported in PROQOLID™ database, we extracted concepts based on the development and/or validation papers of the PROM

After we extracted all concepts, two authors (IS and CA) consolidated the concepts identified into 14 higher-order main concepts to facilitate analysis. These included in alphabetical order: “Belief,” “Compliance,” “Disease activity,” “Health status,” “HRQoL,” “Illness perception,” “Impact (*e.g.*, body image, disruptions at work/school),” “Knowledge and skills,” “Physical functioning (including activities of daily living, disabilities),” “Psychological and cognitive functioning,” “Symptoms,” “Treatment adherence,” “Treatment satisfaction,” and “Utility/quality-adjusted life year.”

We analyzed the data of the abstract/titles of the retrieved articles in two manners:

1. Frequency of mentions: We use the term “mention” to describe the presence of a name/acronym of a PROM in the text of the abstract/title of an included article. Regardless of whether a PROM’s name appeared one time or more than one time in a single abstract/title, this was counted as only one “mention.” A “mention” is not to be confused with an article reference: there were sometimes more mentions than articles because some articles had more than one PROM in their abstract/title.
2. Frequency of use of a PROM: When a PROM was used, the name of the measure was recorded along with the therapeutic indication it was used in, the concept it measured, its category, and its context of use

On the one hand, analyzing the data in terms of the frequency of mentions allowed us to assess the scientific community’s interest in a particular PROM according to the number of articles published about this PROM. On the other hand, analyzing the data in terms of the frequency of use of a particular PROM allowed us to assess how often members of scientific community used this PROM and the ways in which they chose to use it.

##### Results

Out of the 109 selected references, we found 185 mentions of PROMs, 81 therapeutic indications for rare diseases, and 113 PROMs, including two composite measures of PROMs/clinician-reported outcomes. The PROMs most often mentioned were the generic measures *SF-36* (*n* = 21) and *EQ-5D* (*n* = 15). In contrast, we found 47 mentions for rare diseases-specific measures.

**Fig. 3:**
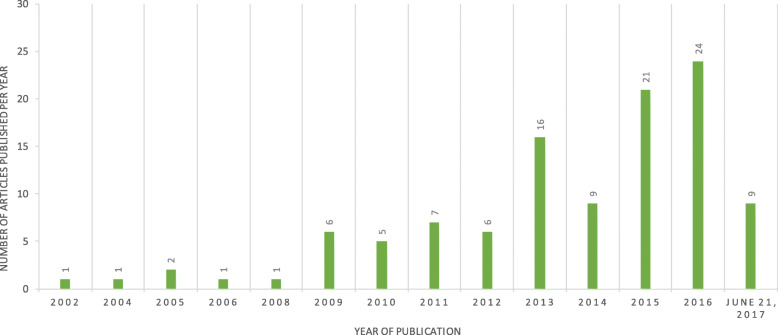
Number of rare disease articles published on PROMs per year

Between 2002 and 2017, the number of articles published per year steadily increased apart from a drop in 2014 (see Fig. 3). Half of the articles were published between 2015 and June 21, 2017. This increasing trend follows with the trend observed in Clinicaltrials.gov study entries.

We used two analytical approaches to identify the most common therapeutic indications (those with 16 or more mentions or 15 or more PROMs) and the most common concepts (those with 4 or more mentions or 6 or more PROMs) assessed in clinical trials. For the first approach, shown in Table 4, we evaluated the frequencies of mentions and the frequencies of PROMs for the most common therapeutic indications; the most common concepts; the four PROM categories (rare disease specific, non-rare disease specific, generic, and study-specific); and the three PROM contexts of use (clinical trial, observational study/registry, and PROM development/validation). For the second approach, we crossed either the frequencies of mentions or the frequencies of PROMs with the frequencies of concepts, categories, and/or contexts of use. Table 5 shows the cross-table of the frequencies of mentions for the contexts of use and the most common concepts. Table 6 shows the cross-table of the frequencies of PROMs for the contexts of use and the PROM categories. Table 7 shows the cross-table of frequencies of PROMs for the PROM categories and the most common concepts.

**Table 4:** Number of mentions and numbers of PROMs for therapeutic indications, concepts measured, PROM category, and PROM context of use

	N = Mention	N = PROM
***Therapeutic indications***		
Hereditary angioedema	13	3
Hunter syndrome	12	6
Rare bone, joint and blood vessel disease	9	9
Systemic sclerosis	8	6
Pulmonary arterial hypertension	7	6
Adiposis dolorosa	6	6
Paroxysmal nocturnal hemoglobinuria	6	6
Ehlers Danlos hypermobile	6	6
Pompe disease	6	5
***Concepts measured by PROMs***		
HRQoL	62	36
Symptoms	47	31
Physical functioning	17	15
Psychological and cognitive functioning	16	15
Other	43	26
***PROM category***		
Generic	72	27
Non-rare disease specific	47	37
Rare disease specific	47	34
Study-specific	19	15
***PROM context of use***		
Observational study/registry	87	61
Clinical trials	66	42
Questionnaire development and validation	32	30

**Table 5:** PROM mentions: Concepts measured in each context of use

Measured quantity: Mention frequency	Number of articles published	HRQoL	Symptoms	Psychological and cognitive functioning	Physical functioning	Other	Total
Observational study/registry	49	34	15	13	8	17	87
Clinical trial	40	19	26	2	4	15	66
Questionnaire development/validation	20	9	6	1	5	11	32
**Total**	**109**	**62**	**47**	**16**	**17**	**43**	**185**

**Table 6:** PROMs: categories used in each context of use

Measured quantity: PROM frequency	Number of articles published	Generic	Non-rare disease	Rare disease specific	Study-specific	Total PROMs
Observational study/registry	49	19	26	9	7	61
Clinical trials	40	12	13	10	7	42
Questionnaire development/validation	20	7	3	18	2	30
**Total**	**109**	**38**	**42**	**37**	**15**	**132**

**Table 7:** PROMs: concepts measured in each category

MEASURED QUANTITY: PROM frequency	HRQOL	Symptoms	Psychological and cognitive functioning	Physical functioning	Other
Generic	9	4	3	6	12
Non-rare disease specific	10	11	10	5	2
Rare disease specific	16	7	2	4	7
Study-specific	1	9	0	0	5
**Total**	**36**	**31**	**15**	**15**	**16**

Regardless of the context of use, PROMs assessing the concepts of “HRQoL” (*n* = 62) and “Symptoms” (*n* = 47) were the most frequently mentioned. The abstract/titles of articles on questionnaire development/validation primarily focused on measures about “HRQoL” (*n* = 9) and secondarily on measures about “Symptoms” (n = 6) and “Physical functioning” (*n* = 5). There was only one mention of an article about the development/validation of a measure for “Psychological and cognitive functioning.” The results for observational studies suggested more focus on the distal and overall impact of diseases: there was more emphasis in these studies on “Psychological and cognitive functioning” PROMs (*n* = 13) and, more generally, on PROMs evaluating “HRQoL” (*n* = 34). These trends contrasted with those observed in the context of clinical trials. Clinical trials mainly employed PROMs to assess proximal impacts and concepts for which a change was likely to occur during the course of a trial. Such concepts were mostly captured with PROMs about “Symptoms” (*n* = 26). That said, 19 mentions of “HRQoL” were also found in the context of clinical trials, reflecting sponsors’ interest in a holistic appraisal of patients’ health and a recognition of the complex nature of rare disease expression.

In the context of clinical trials, generic, rare disease specific, and non-rare disease specific PROMs were used approximately the same number of times. In the context of observational studies/registries, however, a distinction could be found between the different PROM categories: in this context, approximately two-thirds of PROMs (*n* = 45 out of 61) were either generic (*n* = 19) or non-rare disease-specific (*n* = 26). These results suggest that sponsors see observational studies/registries as an opportunity to measure already identified characteristics of diseases. With this goal mind, sponsors choose standard questionnaires, rather than disease-specific PROMs that capture highly specific aspects diseases. In the context of questionnaire development/validation, this tendency was reversed: the overwhelming majority of PROMs were rare disease-specific, which corresponds with sponsors’ investment in developing fit-for-purpose rare disease instruments.

Regardless of the category of the PROM (rare disease specific, non-rare disease specific, generic, or study-specific), the concepts of “HRQoL” and “Symptoms” were dominant. There were in total twice as many PROMs measuring “HRQoL” or “Symptoms” as there were PROMs about “Physical functioning” or “Psychological and cognitive functioning.” About half of the PROMs about “HRQoL” were specific to the rare disease of interest. In contrast, PROMs developed as generic instruments or borrowed from other, non-rare diseases were used to evaluate the concepts of “Physical functioning” and “Psychological and cognitive functioning.” “Symptoms” were generally assessed using specific scales, including study-specific scales.

## Conclusion

### Discussion and conclusion

Over the past 30 years, increased efforts have been made to incorporate the viewpoints of rare disease patients into clinical research. The data presented in this paper reflect this paradigm shift. However, the data also demonstrate that there remains a gap between the importance given to these constituencies and the extent of patient-centricity in orphan drug development. This gap is even more noticeable when we consider that healthcare industry actors commonly use the term “patient-centric” as a buzzword or a catchall phrase. Although sponsors, contract research organizations, and healthcare institutions alike claim to be “patient-centric,” they rarely give a definition of the term “patient-centric” or proof to substantiate their claims. In contrast, in the introduction to this paper, we defined patient-centricity and established clear perimeters for the proof of patient-centricity that we were seeking: the strategic, participatory, and endpoints levels of clinical research in rare diseases.

A search of the literature demonstrates that there is a lack of evidence concerning patient-centricity at the endpoints and strategic levels of clinical research. We could not accurately assess different types of patient engagement, specific contexts in which patient engagement takes place, or precise impacts patient engagement can have. Nonetheless, we could conclude that, now more than in the past, sponsors are asking patients to work hand-in-hand with them on recruitment, retention, and study design. Indeed, there are some encouraging examples in the literature of partnerships between sponsors and patients.

If these examples are to become more common, stakeholders need to have a clear roadmap to follow in the form of a widely adopted industry guidance. Such a guidance would help satisfy two needs. First, the structure that a guidance would give to patient-sponsor interactions would benefit both parties: sponsors would be reassured that patients are reliable partners and patients would be reassured that sponsors are appropriately taking into account their contributions. Second, such a guidance would provide metrics against which to measure patient engagement. These metrics would assist sponsors and patients in identifying successes and areas of improvement.

At the endpoints level, the unique challenges of rare diseases are forcing healthcare industry actors to re-think the strategies they have used to achieve patient-centricity in the context for common diseases. These strategies have proved unsuccessful in the context of rare diseases: the small numbers of rare disease patients, the cognitive and physical capabilities of these patients, and the heterogeneity of rare diseases require that actors develop new strategies. Various regulatory and scientific groups (IRDiRC, FDA, ISPOR Task Force, etc.) are working towards this goal. However, none of these initiatives provides the clear guidance the industry needs. The upcoming release of the FDA’s patient-focused drug development guidance [34] will hopefully address this issue.

Although guidelines to develop and validate a rare disease PROM have not yet been established, the role that PROMs should play in development and approval of rare disease treatments is evident. Using PROMs as endpoints in clinical trials is one of the most important and meaningful ways patients’ experiences can be included and assessed during orphan drug development. However, a review of data on labeling claims, Clinicaltrials.gov entries, and the available literature demonstrates that the use of PROMs in rare diseases is severely lacking and does not show signs of improving.

Rare disease medications are unlikely to have a disease-specific measure in their labeling claim, despite that this is the current practice in the industry for common diseases. Only 17.4% of orphan drugs with a labeling claim from the EMA or the FDA have a PROM in their label. This low percentage may make it difficult for prescribing clinicians to know the impact that authorized orphan medications may have on the HRQoL of their patients. Furthermore, of the 45 orphan medications that do have a PROM in their label, more than half of the PROMs are study-specific symptoms scales, ten are generic PROMs, and only nine are disease-specific, validated scales. The value of study-specific symptoms scales and generic questionnaires may be limited. Symptoms scales may not be developed or validated according to best practice and generic questionnaires are frequently not fit-for-purpose: they are unlikely to undergo cognitive debriefing or psychometric validation within the target population.

The results from the review of the Clinicaltrials.gov entries paint a similarly bleak picture of the place of PROMs in rare disease research. Although there has been a steady increase since 2002 in the number of trials that include a PROM in their study design, less than half of the Clinialtrials.gov entries have a PROM as a primary or secondary outcome. Furthermore, among the PROMs measuring HRQoL used in clinical trials, the majority are generic PROMs and not disease-specific PROMs. Only one study used a PROM as the primary outcome.

The literature review on PROMs in rare diseases reinforces and complements the results from the Clinicaltrials.gov database search. On the one hand, just as with the Clinicaltrials.gov database search results, there is a gradual increase in the number of publications about rare diseases and PROMs from 2002 to 2017. This increase demonstrates a growing interest in tools that capture the patient perspective. On the other hand, several results indicate that outcomes research in rare diseases needs a different solution than the current practice of developing and validating a rare disease-specific measure. In a context where there are now 6,000 to 8,000 identified rare diseases worldwide, and an estimated 250 to 280 rare diseases being discovered per year (1), only 109 articles, 81 therapeutic indications and 34 rare disease specific PROMs were identified in our search results. Furthermore, although there is a positive trend since 2002 in publications on this topic, this trend appears to be too slow to keep pace with the steady rise in the number of rare diseases. Last, despite the fact that most measures found were not specific to the disease of interest, the large variety in the measures we did find complicates the comparability of findings within the same disease and across diseases.

The results indicate that the actual paradigm for developing and using PROMs in rare disease clinical research is failing to meet the needs of patients. Not only are PROMs rarely taken into account in labeling claims or chosen as high-level endpoints in clinical trials, they are also under-researched overall, i.e., there are too few measures for the ever-growing number of rare diseases. Future stakeholders, whether they be sponsors, patients, PAOs, regulatory bodies, or payers, are unlikely to continue to make decisions without having better evidence of patient-perceived benefit. They will demand to know more than how a treatment changes disease clinical parameters or biomarkers. They will want to know about a treatment’s impact on patients’ and their caregivers’ day-to-day lives in terms of symptoms, disease progression, potential side effects, and HRQoL.

There are some ways to address the demands of these constituencies. In the context of clinical trials, patients’ lived experience can be documented using other PCOs besides PROMs, such as observer-reported outcomes and clinician-reported outcomes. Outside of this context, collecting patient preference information is another way to understand how patients and caregivers evaluate benefits and risks [35, 36]. In terms of PROMs, the first step is to consider the feasibility of following the approach suggested in the FDA’S 2009 guidance on patient-reported outcomes development [13]. This was the approach recommended by the ISPOR Task Force [30]. The results we presented demonstrate that researchers face major hurdles when attempting to apply this approach in the context of rare diseases and orphan drug clinical trials. Very few examples exist in orphan drug labels of PROMs developed and used in compliance with this guidance. While some PROMs have been developed that meet regulatory requirements, the time and resources needed for development prevented them from being available early enough to inform regulatory decisions [37, 38]. These delays underscore the need for faster, innovative methods for the development and validation of PROMs for rare diseases that are acceptable to regulators. That said, it is important to recognize that even if such methods could be found, it is not efficient to develop and validate a questionnaire for every one of the known 6000 to 8000 rare diseases [1]. The alternative — choosing to use a generic questionnaire in place of rare disease specific questionnaire — is not an adequate solution either since these generic measures often do not assess concepts important to patients.

We propose a potential solution based on the assumption that stakeholders should assess treatment benefit according to four types of outcomes: (1) disease burden and level of unmet needs, (2) treatment impact and specific hypotheses on drug activity, (3) patient perception of change, and (4) the meaningfulness of perceived change. The methods suggested to assess each of these outcomes are described below:

1. To characterize the disease burden and the level of unmet needs: use generic, well-validated, widely used measures of HRQoL
2. To measure treatment impact and test specific hypotheses on drug efficacy: use well-validated measures, highly specific to a selected body function, for example, hand functioning, visual functioning, and cognitive functioning
3. To assess patient perception of change: use study-specific diaries that capture variations in symptom frequency and severity and the impact of these variations on patients’ daily lives
4. To document meaningfulness of change: ask patients about their individual, personal appraisal of benefits vs. risks using innovative approaches such as qualitative interviewing in the context of clinical trials

The combination of these four types of outcomes and the use of mixed methods will enable sponsors to capture the specificity of a rare disease and to view that disease through a large lens in comparison to other diseases. On the one hand, generic, well-validated questionnaires provide a broad assessment and are helpful for regulators and payers when it comes to market access and pricing. On the other hand, well-validated measures specific to a selected body function serve as a quantitative assessment of the functions that are most important to patients with a specific rare disease. Patient diaries and qualitative interviewing are a useful complement to the quantitative assessments of validated PROMs. Data collected from these sources guarantee a nuanced and personalized assessment of change, which is essential given the variation in rare disease presentation across patients and in the course of a given patient’s life.

## Data Availability

The datasets used and/or analysed during the current study are available from the corresponding author on reasonable request.

## Abbreviations

C-Path: Critical Path Institute
EMA: European Medicines Agency
FDA: United States Food and Drug Administration
HRQoL: Health-related Quality of Life
IRDiRC: International Rare Disease Research Consortium
NICE: National Institute for Health Care Excellence
NORD: National Organization for Rare Disorders
PAO: Patient advocacy organization
PCO: Patient-Centered Outcome
PI: Principal Investigator
PROM: Patient-reported outcome measure
RDCRN: Rare Disease Clinical Research Network
